# An Intriguing Purview on the Design of Macrocyclic Inhibitors for Unexplored Protein Kinases through Their Binding Site Comparison

**DOI:** 10.3390/ph16071009

**Published:** 2023-07-17

**Authors:** Swapnil P. Bhujbal, Jung-Mi Hah

**Affiliations:** 1 College of Pharmacy, Hanyang University, Ansan 426-791, Republic of Korea; swapnil18@hanyang.ac.kr; 2 Institute of Pharmaceutical Science and Technology, Hanyang University, Ansan 426-791, Republic of Korea

**Keywords:** kinases, human diseases, macrocyclic inhibitors, drug discovery

## Abstract

Kinases play an important role in regulating various intracellular signaling pathways that control cell proliferation, differentiation, survival, and other cellular processes, and their deregulation causes more than 400 diseases. Consequently, macrocyclization can be considered a noteworthy approach to developing new therapeutic agents for human diseases. Macrocyclization has emerged as an effective drug discovery strategy over the past decade to improve target selectivity and potency of small molecules. Small compounds with linear structures upon macrocyclization can lead to changes in their physicochemical and biological properties by firmly reducing conformational flexibility. A number of distinct protein kinases exhibit similar binding sites. Comparison of protein binding sites provides crucial insights for drug discovery and development. Binding site similarities are helpful in understanding polypharmacology, identifying potential off-targets, and repurposing known drugs. In this review, we focused on comparing the binding sites of those kinases for which macrocyclic inhibitors are available/studied so far. Furthermore, we calculated the volume of the binding site pocket for each targeted kinase and then compared it with the binding site pocket of the kinase for which only acyclic inhibitors were designed to date. Our review and analysis of several explored kinases might be useful in targeting new protein kinases for macrocyclic drug discovery.

## 1. Introduction

The FDA approval of one of the important drugs, Imatinib, in 2001 marked a milestone in the development of molecularly targeted cancer treatment [1]. It promised the emergence of kinase inhibitors as a crucial drug class in cancer research and other therapeutic areas. There are several reviews reported in the last two decades on the developed kinase inhibitors [1,2]. In this article, we particularized our review of the progress and necessity of macrocyclic inhibitors targeting various protein kinases. There are presently around 71 small-molecule kinase inhibitors (SMKIs) approved by the FDA and additional 16 SMKIs approved by other governing agencies, among which very few are macrocyclic inhibitors [1,2,3]. According to data on SMKI clinical trials, about 110 novel kinases are currently being considered as targets. Together with the approximately 45 targets of approved kinase inhibitors, they represent only about 30% of the human kinome, indicating that there are still extensive unexplored opportunities for this drug class. Hence, we discussed the requisite for the design of more selective and potent macrocyclic kinase inhibitors for the unexplored class of kinases [2].

### 1.1. Need for the Macrocyclic Inhibitors

Macrocycles are typically cyclic modifications of inhibitors resulting from uncyclized classical small molecules or from natural products. In the past few years, macrocycles have drawn progressively more interest in drug discovery due to the several approvals of drug candidates as well as data representing that macrocyclization can improve the biological and physiochemical characteristics in comparison to the acyclic counterparts [3]. Macrocyclic compounds constitute a substantial change in biological activity, molecular shape, and drug-like properties. They have better physicochemical properties, such as good solubility, lipophilicity, metabolic stability, oral bioavailability, increased binding affinity, and overall pharmacokinetics [4,5]. Macrocycles exhibit unique drug-like profiles because of their cyclic nature, conformational and configurational characteristics as well as template-induced preorganization [6,7,8]. Additionally, macrocycles provide a chance for chemical novelty compared to the current scaffolds. For instance, about 90% of the FDA-approved kinase inhibitors are conventional type I/II inhibitors, most of which recurrently share highly similar hinge-binding moieties. After three decades of kinase drug discovery, the chemical space of ATP-mimicking moieties is well studied; however, establishing new hinge-binding moieties can be a major challenge. Consequently, macrocyclization can contribute to the development of a new chemical space for the design of new kinase inhibitors and could be a seamless choice to solve the problem of “undruggable” targets [3,4].

Although macrocyclic compounds have been shown to have therapeutic potential, they have not been fully researched and exploited in drug discovery. Most macrocyclic drugs currently on the market are natural products with complex structures. The complex structure increases the difficulty of synthesis and the cost of production, leading the pharmaceutical industry to be cautious about the development of macrocyclic drugs [9]. Moreover, artificial inhibitors with large ring structures were synthesized and examined for inhibitory activity against several targets. Various studies about ring size effects over the potency of inhibitors were reported earlier [10,11]. Nevertheless, an additional increase in the ring size resulted in a significant reduction in potency [12,13]. Moreover, naturally occurring macrocyclic compounds have ring sizes that span from 11- to 16-membered rings, most frequently 14-membered (Madsen and Clausen 2011) [10]. Hence, the effect of the ring size is very intriguing and can be applicable to studying the interactions obtained by macrocyclic inhibitors with their target [10,12,13]. Some of the naturally occurring macrocyclic compounds are erythromycin (antibiotic), epothilone B (anticancer), tacrolimus (immunosuppressant), and bryostatins (protein kinase C inhibitor) [10].

The most commonly used biological agents have some limitations, including high cost, reduced patient compliance, lack of cell permeability, and low oral bioavailability [14]. Macrocycles are potentially adaptive molecules with enough flexibility to competently interact with flexible binding sites in proteins. Macrocycles have restricted internal bond rotations and are conformationally constrained but not completely rigid [14,15]. Reduction in the overall motion of a receptor, although an unfavorable entropic change, may increase the strength of intermolecular interactions with a ligand, thus increasing favorable enthalpic contribution [15]. These properties of macrocyclic compounds make ‘molecular macrocyclization’ a key means to elucidate the above issues [4,15]. The overall advantage of macrocycles and their expanded chemical diversity benefiting from the advances in synthetic methods. The conformationally restricted macrocyclic compounds need to have an intrinsic shape complementary to the binding site of their target proteins that clarifies their staggered selectivity even for the closely related target molecules [14,15]. The potency of a ligand can either increase or decrease upon macrocyclization based on the specific interactions it has with the protein and the conformations it adopts [4]. Accurate and reliable prediction of the binding affinities of macrocycles to their targets has been published already [16]. These aspects make macrocycles a promising approach for targeting protein–protein interfaces and other shallow or poorly defined binding sites.

### 1.2. Current Development Status of Macrocycles (FDA-Approved Macrocyclic Drugs)

**Figure 1:**
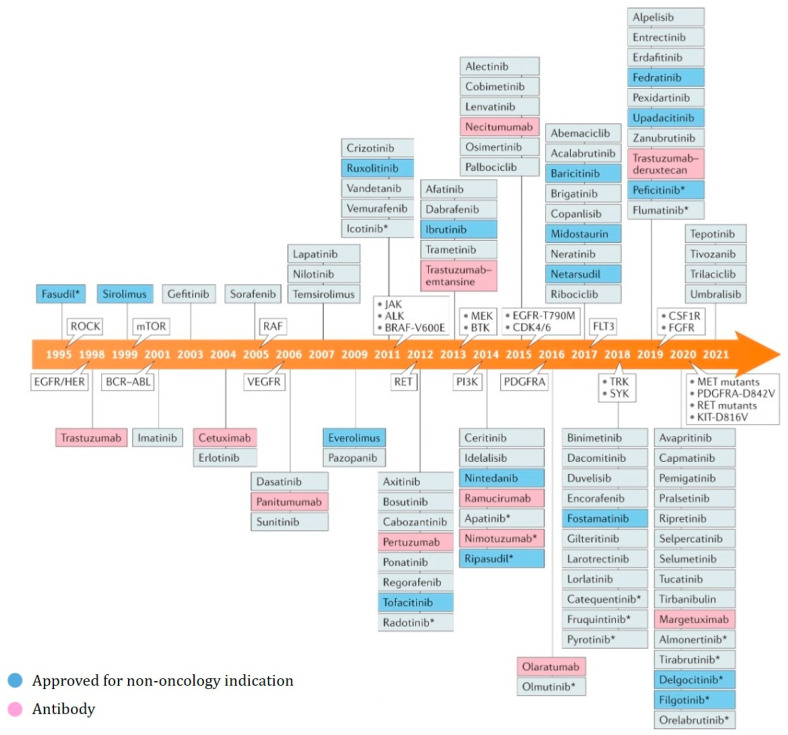
Timeline of approved kinase inhibitors. The timeline shows each small-molecule kinase inhibitor that has been approved since the approval of fasudil in 1995 as well as the year that a novel kinase family was validated (i.e., the first year a drug targeting that kinase family was approved). This figure is copied and modified a bit from reference [2].

Within extensive protein families hosting closely related active sites, achieving selectivity poses a significant challenge. One such family is protein kinases, a prominent target for drug development, comprising over 500 closely related proteins that share a remarkably similar cofactor ATP binding site and overall catalytic domain architecture. Consequently, the orthosteric binding pocket of protein kinases remains highly conserved across this protein family. To address selectivity concerns, the utilization of macrocyclic kinase inhibitors has proven to be a successful approach [3,17]. Figure 1 depicts the timeline of approved kinase inhibitors. The timeline shows each small-molecule kinase inhibitor that has been approved since the approval of fasudil in 1995.

Since 2014, nineteen macrocyclic drugs, including three radiopharmaceuticals, have been approved by FDA for the treatment of bacterial and viral infections, cancer, obesity, immunosuppression, etc. Macrocycles remain an important class of inhibitors and continue to exert a profound influence on chemistry, medicine, and biology [6]. The recent development of macrocyclization has emerged as a strategic approach to improve inhibitor potency and selectivity as well as pharmacokinetic properties. For example, Lorlatinib (PF-06463922) has been developed based on its non-cyclized template, crizotinib [18]. Macrocyclization locked the bioactive conformation of crizotinib, leading to significantly enhanced potency against ALK and ROS and exceptional penetration into the central nervous system. Additionally, lorlatinib is effective against resistance mutations for first- and second-generation ALK inhibitors [17]. In preclinical cell line models, lorlatinib demonstrated potent inhibition of ALK phosphorylation across all ALK mutations, with a 40 to 825-fold improvement in potency compared to crizotinib, an acyclic inhibitor. Notably, PF-06463922 exhibited robust intracranial anti-tumor efficacy in xenograft brain metastasis models, surpassing the effectiveness of both crizotinib and alectinib. In a preclinical pharmacokinetic study involving mice, lorlatinib showed a high concentration in the kidneys, indicating predominant renal elimination. Furthermore, in a phase I open-label trial, concurrent administration of lorlatinib with itraconazole, a potent CYP3A inhibitor, resulted in increased plasma exposure of lorlatinib without any notable increase in serious adverse events. Based on these compelling findings, lorlatinib was identified as a potentially effective treatment for patients with ALK-positive non-small cell lung cancer (NSCLC) while exhibiting an expected safety profile during phase I and phase II trials [19]. BI-4020, an inhibitor of EGFR, serves as another intriguing example of a successful macrocyclization application, which has been designed to target tertiary EGFR resistance mutations, such as EGFR-L858R, EGFR-T790M, and EGFR-C797S that occur in NSCLC [2].

Initially, macrocycles were primarily employed as bioactive compounds or potential medications restricted to natural products. This included rapamycin and its similar rapalog derivatives, presently used as an immunosuppressant or, in the case of temsirolimus, as an oncology drug [3]. Additionally, macrolide antibiotics, exemplified by erythromycin, were discovered nearly 70 years ago and continue to be utilized in the treatment of various bacterial infections [3,4]. Nevertheless, the presence of these initial macrocycles derived from natural sources dissuaded the medicinal chemistry community from incorporating macrocycles into the drug discovery process. This reluctance stems from the fact that macrocycles tend to be large molecules and frequently violate the Lipinski rule of five [3,4]. Moreover, numerous macrocycles derived from natural products feature stereocenters, presenting a significant obstacle to total synthesis and impeding the efficient exploration of structure-activity relationships (SAR) [5].

Numerous synthetic strategies have been devised for the advancement of macrocyclic kinase inhibitors [3], and few of these macrocycles have received drug approval or entered clinical trials that, as shown in Table 1 [2,3]. However, significant progress has recently been made to simplify the synthesis of macrocyclic compounds that can be synthesized using ring-closing metathesis [3,20]. A detailed study/description of the synthesis of macrocycles has been published somewhere else [2,3,4,6].

#### FDA-Approved Macrocyclic Drugs

The macrocyclic drugs approved by the FDA for marketing are stated in Table 1 and are discussed below consecutively.

Sirolimus (1), also known as rapamycin, is a macrocyclic lactone antibiotic produced by the bacteria Streptomyces hygroscopicus. Originally, it was isolated and identified as an antifungal compound. However, upon the subsequent discovery of its potent antitumor and immunosuppressive properties, extensive research focused on exploring its use as an immunosuppressive and antitumor agent. The primary mode of action involves the inhibition of the mammalian target of rapamycin (mTOR), a protein kinase belonging to the serine/threonine kinase family that plays a crucial role in regulating cell growth, proliferation, and survival. Sirolimus obtained its first FDA approval in 1999 for the prophylaxis of organ rejection in patients aged 13 years and older who underwent renal transplants. The European Agency acknowledged sirolimus as an alternative to calcineurin antagonists for maintenance therapy in November 2000. Subsequently, in May 2015, the FDA granted approval for sirolimus in the treatment of patients with lymphangioleiomyomatosis. Furthermore, in November 2021, the FDA approved albumin-bound sirolimus for intravenous administration to treat adults diagnosed with metastatic malignant perivascular epithelioid cell tumor (PEComa) [2,3,6].

Temsirolimus (2) is an antineoplastic agent employed in the treatment of renal cell carcinoma (RCC), applying its therapeutic effects through mTOR inhibition. Derived from sirolimus, Temsirolimus has been specifically developed for the treatment of RCC and was brought to fruition by Wyeth Pharmaceuticals, marketed under the trade name Torisel. In late May 2007, Temsirolimus received FDA approval, followed by approval from the European Medicines Agency (EMEA) in November 2007 [2,3].

Everolimus (3) is a derivative of Rapamycin (sirolimus) and exerts its actions in a manner similar to Rapamycin as an inhibitor of mTOR. Presently, Everolimus is utilized as an immunosuppressant against organ transplant rejection [2,3].

Lorlatinib (4), a third-generation ALK tyrosine kinase inhibitor (TKI), received its initial FDA approval in November 2018 as a treatment for ALK-positive metastatic non-small cell lung cancer. Subsequently, in 2019, the EMA also approved Lorlatinib for the treatment of certain previously treated patients with advanced ALK-positive non-small cell lung cancer. Furthermore, in 2022, the approval was expanded to include Lorlatinib as a first-line treatment option for advanced ALK-positive NSCLC. Significantly, Lorlatinib exhibits remarkable penetration into the central nervous system (CNS) and demonstrates high selectivity towards its intended targets, ROS and ALK, in comparison to its noncyclized template R-Crizotinib. The compelling instances of Lorlatinib and BI-4020 provided clear evidence that by locking the bioactive conformation of linear precursor molecules, significant enhancements were achieved in target potency, selectivity across the kinome, and crucial physicochemical and in vivo properties such as brain penetration [2,3,6].

Pacritinib (5), an inhibitor of both wild-type and mutant (V617F) JAK2 as well as FMS-like tyrosine kinase 3 (FLT3), is employed in the treatment of primary and secondary myelofibrosis (MF) in adult patients with significantly weakened platelet counts. In February 2022, Pacritinib was granted accelerated approval by the FDA for the treatment of both primary and secondary myelofibrosis. This approval offers a treatment alternative for patients with MF and severe thrombocytopenia, a condition observed in approximately one-third of MF patients and associated with an especially unfavorable prognosis [2,3,6].

E6201 (ER-806201) (6) is an ATP-competitive dual kinase inhibitor of MEK1 and FLT3, demonstrating efficacy in both anti-tumor and anti-psoriasis applications [2,3].

Zotiraciclib (TG02) (7) is a potent CDK/JAK2/FLT3 inhibitor, which is under investigation in clinical trial NCT02942264 (randomized Phase 2 trial in adults with Recurrent Anaplastic Astrocytoma and Glioblastoma) [2,3].

Selitrectinib (LOXO-195) (8) is a next-generation TRK kinase inhibitor. It is under investigation in clinical trial NCT03215511 (Phase 1/2 study of LOXO-195 in patients with previously treated NTRK fusion cancers) [2,3,9].

Repotrectinib (TPX-0005) (9) is a novel ALK/ROS1/TRK inhibitor as well as a potent SRC inhibitor. According to Turning Point Therapeutics, Inc., the FDA has granted breakthrough therapy designation to repotrectinib for the treatment of patients diagnosed with ROS1-positive metastatic non-small cell lung cancer (NSCLC). This designation applies specifically to patients who have previously undergone treatment with a ROS1 tyrosine kinase inhibitor and have not received platinum-based chemotherapy before [2,3].

SB1578 (10) is a novel, orally bioavailable JAK2 inhibitor with specificity for JAK2 within the JAK family and also potent activity against FLT3 and c-Fms. The activation of these three tyrosine kinases is crucial in the pathways involved in the development of rheumatoid arthritis. SB1578 effectively hinders the activation of these kinases, along with their subsequent signaling in relevant cells. This inhibition ultimately leads to the suppression of pathological cellular responses associated with the condition [2,3].

JNJ-26483327 (11), also known as BGB102, is a small-molecule reversible tyrosine kinase inhibitor that is orally bioavailable and exhibits potential as an antineoplastic agent. Acting in a multitargeted manner, BGB102 binds to and inhibits various members of the epidermal growth factor receptor (EGFR) family, including EGFR, HER2, and HER4. Additionally, it targets Src family kinases (Lyn, Yes, Fyn, Lck, and Src) and vascular endothelial growth factor receptor type 3 (VEGFR3) [2,3,6].

More details about these drugs can be found in the references [2,3,5,6].

### 1.3. Previously Explored Various Kinase Targets

Kinases have an essential role in regulating intracellular signaling pathways that control crucial cellular processes such as cell proliferation, survival, differentiation, and apoptosis. Deregulation of kinase activity has been implicated in over 400 diseases [17]. For instance, mutations in genes encoding protein kinases can lead to excessive kinase activity. Several clinically approved drugs, such as selective kinase inhibitors, are designed to target and inhibit the enzymatic activity of specific protein kinases. These drugs are employed in the treatment of various conditions, including Alzheimer’s disease, polycystic kidney disease, rheumatoid arthritis, cancer, and numerous others [21]. FDA-approved protein kinase inhibitors, along with their respective targets, are listed in Figure 1, which includes acyclic as well as cyclic inhibitors. 

**Figure 2:**
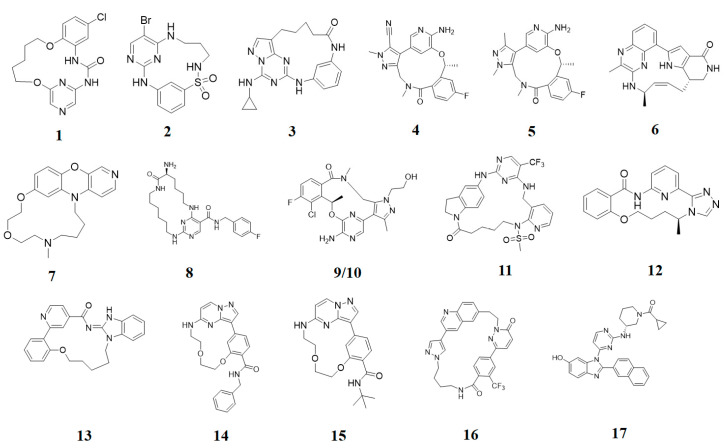
2D-Structures of co-crystallized macrocyclic inhibitors with their respective protein kinases discussed above.

The previous Section 1.2 already covers the macrocyclic inhibitors that have been approved for various kinases. Therefore, in this section, we will focus on the kinases that are currently being targeted for the development of macrocyclic compounds or the target proteins that have a 3D structure co-crystallized with macrocyclic compounds available on the protein data bank (PDB): “URL: https://www.rcsb.org/” (accessed on 25 May 2023). Totally, 16 structures meeting these criteria were obtained and used to compare their binding site pocket with the binding site of unexplored kinases. The information regarding these structures was extracted from the protein data bank (PDB) and their respective literature, which is summarized below:

**Proline-rich tyrosine kinase 2 (Pyk2)** belongs to the focal adhesion kinase (FAK) subfamily and is a non-receptor cytoplasmic tyrosine kinase. Both Pyk2 and FAK play important roles in various signaling pathways that govern cell migration, proliferation, and survival. In recent years, studies involving the genetic knockdown of Pyk2 and the use of small molecule inhibitors have highlighted the potential therapeutic significance of Pyk2 in osteoporosis treatment. The catalytic domains of Pyk2 and FAK kinases share a sequence similarity of 73%. The residues responsible for forming the ATP binding sites of Pyk2 and FAK exhibit a slightly higher similarity of 78%. As a result, the search for a selective small molecule inhibitor specifically targeting Pyk2 has proven to be challenging. To address this, Farand et al. directed their attention towards diaminopyrimidine-based inhibitors PF-431396 and PF-562271, ultimately developing **compound 11** (as shown in the Figure 2 below, **hereon**) (7-(methylsulfonyl)-3^5^-(trifluoromethyl)-2,4,7-triaza-1(5,1)-indolina-3(2,4)-pyrimidina-6(3,2)-pyridinacyclododecaphan-12-one), which was co-crystallized with the receptor Pyk2 (PDB ID: 5TO8) [22].

**Pim-1**, -2, and -3 are a closely related group of serine/threonine kinases belonging to the CAMK (calmodulin-dependent kinase) family. The observation of elevated expression of Pim-1/2 kinases in B-cell malignancies indicates that inhibitors targeting Pim kinases could be beneficial in treating lymphoma, leukemia, and multiple myeloma. Beginning with a moderately potent quinoxalinedihydropyrrolopiperidinone lead compound, the potential for macrocyclization was recognized, leading to the development of a series of 13-membered macrocycles. This effort resulted in the discovery of a potent **inhibitor**, **6** ((2^7^S,6R,Z)-1^3^,6-dimethyl-2^4^,2^5^,2^6^,2^7^-tetrahydro-2^1^H-7-aza-1(8,2)-quinoxalina-2(2,7)-pyrrolo [3,2-c]pyridinacycloheptaphan-4-en-2^4^-one) that was co-crystallized with the receptor Pim1 (PDB ID: 5EOL) [23].

Similarly, another **Pim-1** co-crystal structure was reported (PDB ID: 7XSV) with **compound 7** (8-methyl-1^5^H-2,5-dioxa-8-aza-1(7,5)-benzo[b]pyrido [4,3-e][1,4]oxazinacyclododecaphane) [24]

**Checkpoint kinase 1 (Chk1)** is a serine/threonine protein kinase that plays a crucial role in the DNA damage-induced checkpoint network and the normal cell cycle. Inhibiting Chk1 disrupts the S and G2 checkpoints, sensitizing tumor cells, particularly those lacking the p53 gene, more susceptible to different DNA-damaging agents. The referenced study delves deeper into the exploration of urea-based inhibitors targeting Chk1 kinase within a macrocyclic ring system, ultimately resulting in the discovery of **compound 1** (5^5^-chloro-6,12-dioxa-2,4-diaza-1(2,6)-pyrazina-5(1,2)-benzenacyclododecaphan-3-one) that was co-crystallized with Chk1 (PDB ID: 2E9U) [14,25].

**Cyclin-dependent kinases (CDKs)** are a type of serine/threonine kinases that depend on the association with a cyclin regulatory subunit to become active. They play a crucial role in organizing the proper timing and sequence of events during the cell division cycle. Dysregulated control of CDKs and the subsequent loss of cell-cycle checkpoint function have been linked to the molecular mechanisms underlying cancer. X-ray structure (PDB ID: 2J9M) of macrocyclic aminopyrimidine **2** (1^5^-bromo-4-thia-2,5,9-triaza-1(2,4)-pyrimidina-3(1,3)-benzenacyclononaphane 4,4-dioxide) in complex with CDK2 revealed significantly improved inhibitory properties (IC_50_ = 20 nM) [14,26].

**Protein kinase CK2** is an extensively conserved and pleiotropic serine/threonine kinase that is pivotal in cellular growth, proliferation, and survival. Notably, CK2 has been consistently found to be overexpressed in a diverse range of human cancers. A series of macrocyclic derivatives were developed based on the X-ray co-crystal structures of pyrazolo [1,5-a] [1,3,5]triazines with corn CK2 (cCK2) protein. Bioassays demonstrated that these macrocyclic pyrazolo [1,5-a] [1,3,5]triazine compounds (**Compound 3** ((1^1^Z,1^8^Z)-1^4^-(cyclopropylamino)-2,4-diaza-1(2,8)-pyrazolo [1,5-a][1,3,5]triazina-3(1,3)-benzenacyclononaphan-5-one)) were potent CK2 inhibitors and strongly inhibit cancer cell growth (PDB ID: 3BE9) [27].

**Anaplastic lymphoma kinase (ALK)** belongs to the insulin receptor (IR) kinase subfamily. It is predominantly expressed in adult brain tissue and plays a significant role in the development and proper functioning of the nervous system. The designation “ALK” stems from its identification as a crucial driver in anaplastic large-cell lymphoma (ALCL). The crystal structure of ALK with **Lorlatinib** (4) described for the first time has been deposited in the protein data bank (PDB ID: 4CLI) [3,28].

Similarly 3D structure of another macrocyclic **compound 5** ((R)-2^6^-amino-5^5^-fluoro-1^1^,1^3^,4,7-tetramethyl-1^1^H-3-oxa-7-aza-2(3,5)-pyridina-1(4,5)-pyrazola-5(1,2)-benzenacyclooctaphan-6-one) co-crystallized with ALK (PDB ID: 4CMU) was available [28].

**The epidermal growth factor receptor (EGFR)** is a receptor tyrosine kinase responsible for transmitting mitogenic signals. Genetic mutations within the EGFR gene are detected in around 12–47% of non-small cell lung cancer (NSCLC) tumors characterized by adenocarcinoma histology. **Compound 13** ((*E*)-5^2^,5^3^-dihydro-5^1^H-11-oxa-4-aza-5(2,1)-benzo[d]imidazola-2(2,4)-pyridina-1(1,2)-benzenacycloundecaphan-3-one) from this reference was co-crystallized with EGFR (PDB ID: 6S9D) [4,29].

**Tyro3, Axl, and Mer (TAM)** form a receptor tyrosine kinase family that has been relatively recently discovered. This family of kinases plays a crucial role in immune responses. However, Axl, in particular, has also been associated with cancer; thus, it became a target of interest in the search for new therapeutic interventions. The 3D structure of **compounds 9 and 10** ((R)-2^5^-amino-5^6^-chloro-5^5^-fluoro-1^1^-(2-hydroxyethyl)-1^3^,4,7-trimethyl-1^1^H-3-oxa-7-aza-2(2,6)-pyrazina-1(4,5)-pyrazola-5(1,2)-benzenacyclooctaphan-6-one) was reported with AXL and Mer kinase, respectively. (PDB ID: 5U6B and 5U6C) [30].

**c-Met,** a distinctive member of the receptor tyrosine kinase (RTK) subfamily, functions as the receptor for hepatocyte growth factor (HGF)/scatter factor (SF). Small molecule inhibitors targeting c-Met are presently the subject of extensive research and hold significant therapeutic promise for the treatment of non-small-cell lung cancer (NSCLC). Thus, Wang et al. reported the co-crystal structure of macrocycle (16) ((1^4^Z,5^2^E)-6^3^-(trifluoromethyl)-5^1^,5^6^-dihydro-1^1^H-8-aza-2(3,6)-quinolina-5(1,3)-pyridazina-1(4,1)-pyrazola-6(1,4)-benzenacyclododecaphane-5^6^,7-dione) with c-Met (PDB: 8GVJ) [31].

**Serine/threonine kinase 17A**, also known as death-associated protein kinase-related apoptosis-inducing protein kinase 1 (**DRAK1**), is a member of the death-associated protein kinase (DAPK) family and falls within the category of the “dark kinome.” DRAK1 has been implicated in the development of glioblastoma multiforme (GBM) and other types of cancers. However, there are currently no specific inhibitors that selectively target DRAK1. Hence, Kurz et al. optimized a pyrazolo [1,5-a]pyrimidine-based macrocyclic scaffold, and their structures were deposited in PDB as follows: **Compound 14** ((1^3^Z,1^4^E)-N-benzyl-3,6-dioxa-9-aza-1(3,5)-pyrazolo [1,5-a]pyrimidina-2(1,3)-benzenacyclononaphane-2^4^-carboxamide) (PDB ID: 7QUE) and **Compound 15** ((1^3^Z,1^4^E)-N-(tert-butyl)-3,6-dioxa-9-aza-1(3,5)-pyrazolo [1,5-a]pyrimidina-2(1,3)-benzenacyclononaphane-2^4^-carboxamide) (PDB ID: 7QUF) [32].

**Apoptosis signal-regulating kinase 1** (ASK1, MAP3K5) is a member of the mitogen-activated protein kinase kinase kinase (MAP3K) family, and it plays a pivotal role in the cellular stress response by regulating inflammation and apoptosis. These two kinases are critical in initiating the inflammatory and apoptotic stress responses. Modulating the activity of ASK1 could have implications for the treatment of neurological disorders such as amyotrophic lateral sclerosis (ALS), multiple sclerosis (MS), Parkinson’s disease (PD), and Alzheimer’s disease (AD). The 3D structure of ASK1 with **compound 12** ((*S*)-10-methyl-1^4^H-6-oxa-3-aza-2(2,6)-pyridina-1(3,4)-triazola-5(1,2)-benzenacyclodecaphan-4-one) have been reported (PDB ID: 6OYW) [33].

Using a structure-based drug design strategy, macrocyclic pyrimidines were developed as highly potent inhibitors targeting the **Mer tyrosine kinase (MerTK).** Extensive SAR (structure-activity relationship) investigations revealed that analog 8, also known as UNC2541, emerged as a crucial compound with remarkable sub-micromolar inhibitory potency. Additionally, an X-ray structure of MerTK in complex with **compound 8** ((*S*)-7-amino-N-(4-fluorobenzyl)-8-oxo-2,9,16-triaza-1(2,4)-pyrimidinacyclohexadecaphane-1^5^-carboxamide) was resolved to show that these macrocycles bind in the MerTK ATP pocket (PDB ID: 5K0X) [10].

The 2D structures of ligands and each selected protein, along with its PDB ID, are presented in Table 2.

### 1.4. Unexplored Targets for Macrocyclic Inhibitors

The family of kinases poses three significant challenges in the process of drug design [4]. Firstly, inhibitors must possess high potency, typically within the low nanomolar range, in order to effectively compete with the abundant cellular concentrations of ATP. Secondly, a high level of specificity is necessary to selectively target the desired kinase while avoiding interference with other kinases within this extensive enzyme family, thereby reducing the risk of side effects. Lastly, these requirements need to be fulfilled while maintaining the physicochemical properties of the designed compounds that are favorable for further development, often referred to as drug-like properties [21]. Using a macrocyclic strategy in the design of kinase inhibitors presents a compelling and innovative solution to address these challenges. Macrocyclic kinase inhibitors are characterized by their relatively small molecular weight and their ability to bind to the hinge region of kinases, akin to ATP. The conformationally constrained 3D structure of these inhibitors offers an optimal shape that complements the ATP binding site, resulting in enhanced potency and selectivity [22,23,25,27,28,29,30,31,32,33].

In contrast to the flexible linear type 1 inhibitors, macrocycles possess the capability to exhibit exceptional selectivity, even towards closely related subfamily members of kinases, by detecting subtle shape variations in the binding pocket. Furthermore, the compact size of macrocycles confers an additional advantage, as their physicochemical properties align more favorably with the subsequent development of potent drug candidates [34,35]. The relatively low molecular weight of these compounds, which is typically around half of that of a conventionally optimized linear kinase inhibitor, offers significant benefits in terms of pharmacokinetic properties. Moreover, this reduced size has the potential to facilitate their ability to penetrate the blood-brain barrier [21,22]. Macrocyclic compounds have been developed and reported as promising drug candidates for various diseases, with a particular emphasis on their therapeutic applications in the field of oncology [34,35,36]. Therefore, it is advisable to develop novel macrocyclic inhibitors targeting kinases for which only non-cyclic inhibitors had been previously documented, for example, JNK3 (c-Jun N-terminal kinase 3).

JNKs are a family of stress-activated serine-threonine protein kinases belonging to the mitogen-activated protein kinases (MAPKs) [37]. They are involved in the regulation of many cellular activities, from proliferation to cell death [37]. JNK1 and JNK2 are widely expressed in all body tissues. However, JNK3 is expressed only in the central nervous system (CNS), cardiac, smooth muscle, and testis [38]. It is mainly involved in neurodegenerative processes like Alzheimer’s disease (AD), Parkinson’s disease (PD), cerebral ischemia, and other CNS disorders. Notably, JNK3 was detected in the cerebrospinal fluid (CSF) of AD patients, and its increased level is statistically correlated with the rate of cognitive decline, indicating that JNK3 is a key player in this disease, which makes JNK3 an attractive CNS drug target [37,38]. Furthermore, JNK3 plays a key role in the first neurodegenerative event, the perturbation of physiological synapse structure and function, known as synaptic dysfunction. Synaptic dysfunction and spine loss have been reported to be pharmacologically reversible, opening new therapeutic directions in brain diseases. JNK3 could be used as a disease biomarker as it is detectable at the peripheral level, allowing an early diagnosis of neurodegenerative and neurodevelopment diseases in a still prodromal stage [37,38,39].

At present, it seems that the development of inhibitors for a minimum of 11 kinase families and around 20 kinase targets has been discontinued [1,2,38]. The absence of agents in active trials and the lack of recent status reports, some of which are dated over five years ago, indicate that these projects have been abandoned [2]. An example of one such kinase is the Jun N-terminal kinase (JNK) family, which plays a crucial role in regulating cell survival and proliferation in response to cytokines and growth factors. JNKs have been implicated in various diseases, including cancer, immune disorders, and neurodegenerative conditions. Clinical trials were initiated for four JNK inhibitors targeting myeloid leukemia (CC-401; phase I) in 2005, fibrotic disorders (CC-930; phase II) in 2011, endometriosis (PGL5001; phase II) in 2012, and hearing loss (AM-111; phase III trial completed in 2017) [1,2]. Unfortunately, none of these inhibitors have advanced beyond their respective trial stages. Possible explanations for the limited progress of these inhibitors include commonly encountered issues such as toxicity and insufficient specificity toward their target [2,3].

Given this information, it is worth considering JNK3 as an illustrative example since it has not been extensively studied as a target for the development of macrocyclic inhibitors in the past. Targeting unexplored protein kinases like JNK3 could prove advantageous in the design and advancement of macrocyclic inhibitors for the treatment of diverse cancer types and neurodegenerative disorders.

## 2. Binding Site Comparison

The three-dimensional (3D) structure of proteins is of utmost significance to understand the biological functions of proteins [40]. Comparing the binding sites of various proteins offers valuable insights into the process of drug discovery and development. Such comparisons can aid in understanding polypharmacology, identifying potential off-targets, and repurposing existing drugs [40]. A variety of computational techniques exist to assess protein structures for tackling diverse scientific challenges [41,42]. Among these, several computational methods have been developed specifically for comparing ligand binding sites in protein structures. These methods can be broadly categorized into three groups: residue-based, surface-based, and interaction-based methods. For detailed explanations of these methods, refer to other sources [43]. The increasing number of available binding site comparison methods presents a challenge in selecting the most suitable method for a particular research area.

### 2.1. SiteMap

In this study, we utilized SiteMap [44] package available in Schrödinger Maestro 13.1 (Release 2022-1, Schrödinger, LLC, New York, NY, USA) to compare the binding sites of multiple proteins (X-ray crystal structures bound to their respective co-crystallized ligands listed in Table 2) with the binding site of JNK3. SiteMap employs innovative search and analysis capabilities to provide valuable insights into the nature of binding sites. The calculation process involves three stages. Firstly, an initial search identifies one or more regions, known as sites, located on or near the protein surface that have the potential to bind a ligand. Subsequently, site maps are generated, consisting of hydrophobic and hydrophilic maps. The hydrophilic maps are further subdivided into the donor, acceptor, and metal-binding regions. During the evaluation stage, the SiteMap calculation analyzes each site by computing diverse properties, such as the volume of the binding pocket. The generated site maps can assist in the design of improved ligands by identifying “targets of opportunity” [44,45]. These targets may include hydrophobic regions with sufficient space to accommodate larger hydrophobic groups, presenting potential opportunities for ligand optimization. Furthermore, site maps have multiple applications, including the selection of a target for ligand docking using Glide and the evaluation of docking hits. They provide valuable insights into the extent to which docking poses exhibit appropriate complementarity to the receptor. The regions that do not exhibit hydrophobic or hydrophilic characteristics hold significance as they indicate potential areas where the physical properties of the ligand can be enhanced, such as solubility, with minimal impact on the binding affinity. In contrast to techniques that employ color-coded representations of hydrophilicity or hydrophobicity on the receptor surface, site maps consider the overall nature of the site rather than solely focusing on the nearest receptor atom. Additionally, site maps explicitly depict the shape and boundaries of both philic and phobic regions, offering a level of detail that surface-based displays are unable to provide.

We aimed to investigate whether the similarity in the binding site could explain the presence of sufficient binding pocket area for the design of macrocyclic compounds. To accomplish this objective, we calculated the binding pocket volume for each selected structure (complex) and compared it to the binding pocket volume of JNK3.

### 2.2. Comparison of Protein Binding Site

**Figure 3:**
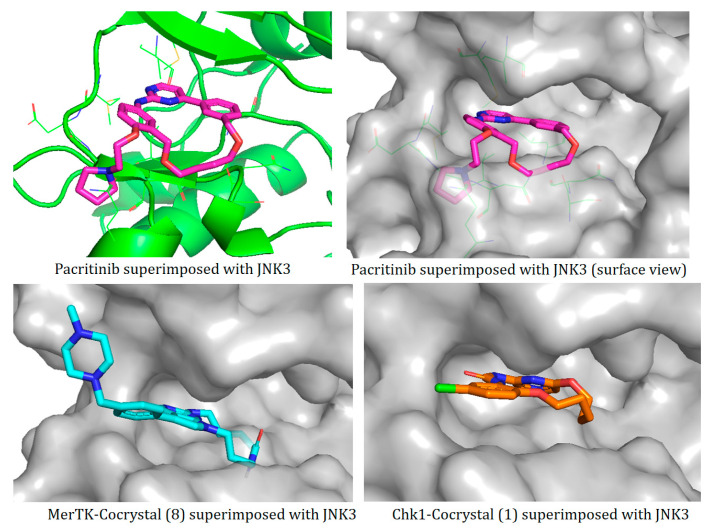
Superimposition of a few existing macrocyclic inhibitors inside the binding pocket of JNK3 (PDB: 4KKH).

We have employed the SiteMap [44] calculations to compare the binding pocket of JNK3 with the binding pocket of kinases for which macrocyclic inhibitors have been designed before (as shown in Table 2, performed by us). The final column in Table 2 illustrates the binding pocket of each kinase, representing the depth of the pocket using white balls. The sitemaps were intentionally hidden to provide a clearer view of the binding pocket. There are around 16 protein kinase structures co-crystallized with their respective macrocyclic compounds available on the protein data bank (PDB). To validate our hypothesis, we selected these kinases and compared their binding pockets with that of JNK3, considering variations in volume. The binding pockets of all the chosen kinases (listed as 1–16 in Table 2) were compared to the binding pocket of JNK3 (listed as 17 in Table 2). Through the calculation of the binding pocket volume for each kinase, we observed that JNK3 consistently exhibited an equivalent or larger pocket size (341.285 (Å)^3^) than the other kinases except for CDK2 and CK2, which possess a volume of (384.160 (Å)^3^) and (365.638 (Å)^3^), respectively. The rest of the kinases showed a binding pocket volume less than that of the binding pocket volume of JNK3. Furthermore, we conducted superimposition analyses involving the smallest (Chk1 compound 1), largest (MerTK compound 8) co-crystal structures, and Pacritinib within the binding pocket of JNK3. The aim was to assess whether these inhibitors could effectively occupy the available space in the active site pocket. Figure 3 illustrates the results, demonstrating that all three superimposed inhibitors could easily fit into the binding pocket of JNK3.

Based on our analysis, it reveals that JNK3 possesses the ability to accommodate new macrocyclic inhibitors and has the potential to establish significant interactions with its amino acid residues. Conducting a more extensive examination of the binding of existing macrocyclic inhibitors with JNK3 could provide valuable insights into the key interaction sites within the JNK3 active site. Moreover, by considering the accessibility of the binding site pocket and the critical interaction hotspots, medicinal chemists can determine the optimal size of macrocycles specifically to target JNK3 or other protein kinases with improved physicochemical properties. This perspective would undoubtedly facilitate the targeting of novel protein kinases for the design of macrocyclic inhibitors in a similar manner.

## 3. Conclusions

Macrocyclic kinase inhibitors have exhibited enhanced properties when compared to their acyclic counterparts, displaying promise for the development of novel drug candidates. However, it is important to note that macrocyclization alone does not guarantee the creation of optimal bioactive kinase inhibitors suitable for clinical use. In addition to factors such as selectivity and potency, structural modifications can also affect physicochemical features, safety profiles, as well as pharmacodynamic and pharmacokinetic properties. Furthermore, through careful evaluation of the accessibility of the binding site pocket and identification of critical interaction hotspots, we can determine the ideal size of macrocycles to target JNK3 or other protein kinases. This approach enables the development of macrocycles with enhanced physicochemical properties, offering potential benefits in drug design and optimization. Hence, it is crucial to subject novel molecules generated through macrocyclization techniques to thorough optimization using relevant in vitro and pharmacokinetic/pharmacodynamic models before progressing to late preclinical studies. Currently, few macrocyclic kinase inhibitors have received FDA approval or are undergoing evaluation in clinical trials for diverse therapeutic indications. As a result, macrocyclization represents a valuable approach for the CADD community and medicinal chemists in their pursuit of developing innovative therapeutic and diagnostic agents for human diseases.

## Figures and Tables

**Table 1:** FDA-approved macrocyclic kinase inhibitors and inhibitors in clinical trials.

No.	Structure	Name	Target(s)	Sponsor	Status (Clinical Trial)
1	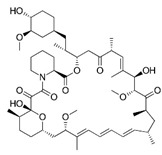	Sirolimus (Rapamycin)	mTOR	Wyeth/Pfizer	FDA approved
2	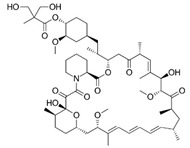	Temsirolimus		Wyeth/Pfizer	FDA approved
3	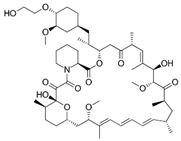	Everolimus		Novartis	FDA approved
4	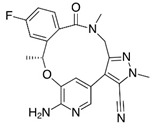	Lorlatinib (PF-06463922)	ROS, ALK	Pfizer	FDA approved
5	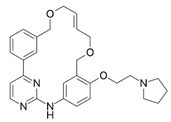	Pacritinib (SB1518)	JAK2, FLT3	CTI Bio Pharma	FDA approved
6	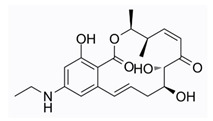	E6201	MEK1, FLT3	Eisai Inc.	Phase 2
7	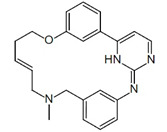	Zotiraciclib (TG02; SB1317)	CDK2, JAK2, FLT3	National Cancer Institute (NCI)	Phase 2
8	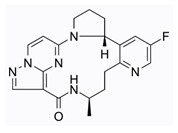	Selitrectinib (LOXO-195)	TRKA, TRKB, TRKC	Bayer	Phase 1/2
9	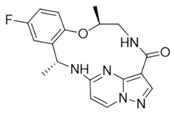	Repotrectinib (TPX-0005)	TRKA, TRKB, TRKC, ROS1, ALK	Turning Point Therapeutics. Inc.	Phase 1/2
10	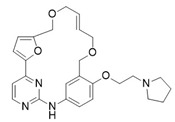	SB1578	JAK2	S*Bio	Phase 1
11	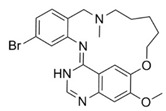	JNJ-26483327	EGFR, RET, VEGFR-3, Her4, Lyn, Fyn, Yes	Johnson & Johnson	Phase 1

**Table 2:** Binding pocket volume calculation of different protein kinases and 2D structures of their bound macrocyclic compounds.

No	Protein Name	PDB ID	Volume of Binding Pocket (Å)^3^	2D Structure of Co-Crystallized Ligand	Binding Pocket of Protein Shown with Its Co-Crystallized Ligand
1	Chk1	2E9U	259.994	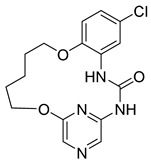	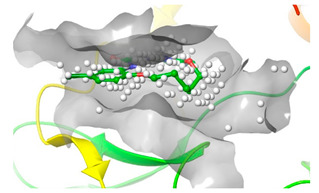
2	CDK2	2J9M	384.160	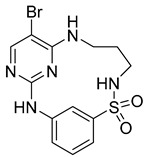	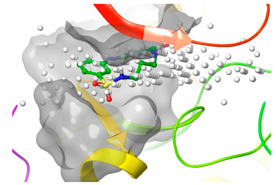
3	CK2	3BE9	365.638	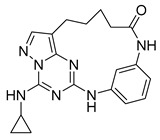	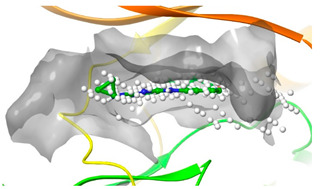
4	ALK	4CLI	263.081	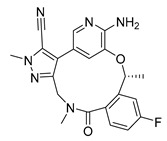	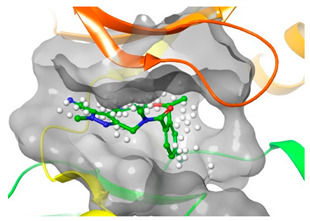
5	ALK	4CMU	238.042	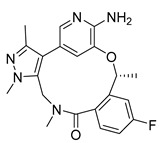	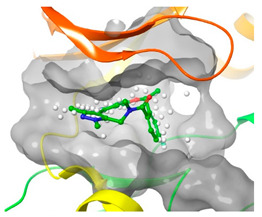
6	Pim1	5EOL	309.729	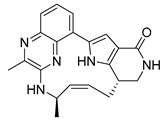	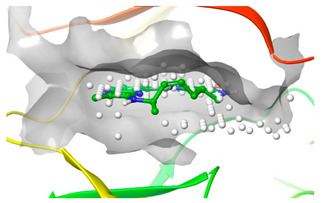
7	Pim1	7XSV	242.158	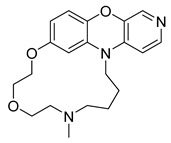	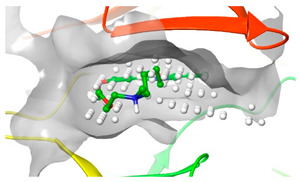
8	MerTK	5K0X	292.236	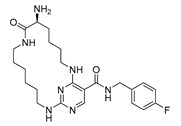	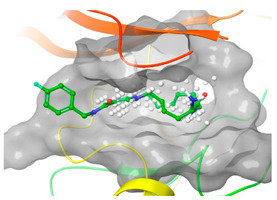
9	MerTK	5U6C	260.337	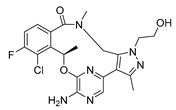	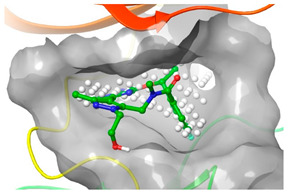
10	AXL	5U6B	293.951		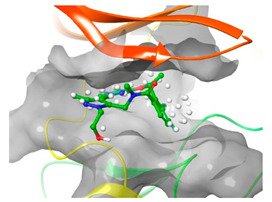
11	PYK2	5TO8	280.231	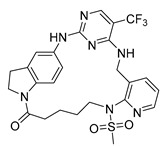	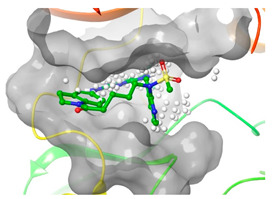
12	ASK1	6OYW	238.728	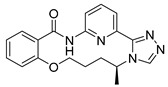	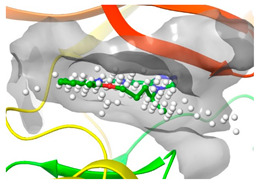
13	EGFR	6S9D	293.265	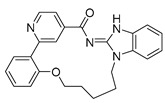	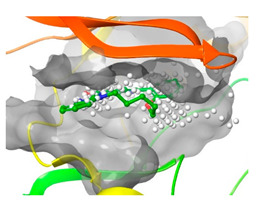
14	DRAK1	7QUE	318.647	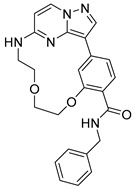	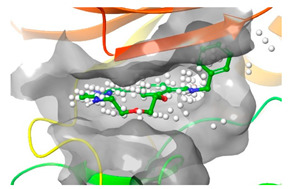
15	DRAK1	7QUF	311.444	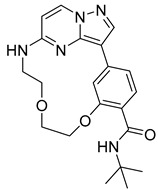	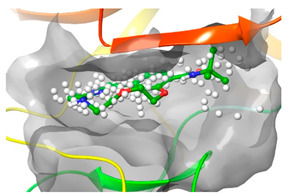
16	cMET	8GVJ	251.076	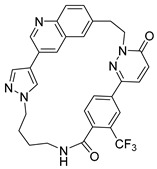	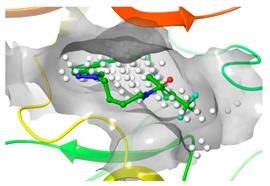
**17**	**JNK3**	**4KKH**	**341.285**	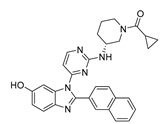	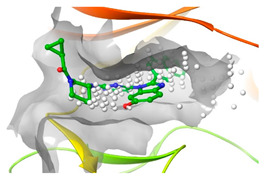

## Data Availability

Data sharing not applicable.

## References

[1] Zimmermann J. (2009). Interview with Jürg Zimmermann, global head of oncology & exploratory chemistry at Novartis. Future Med. Chem..

[2] Attwood M.M., Fabbro D., Sokolov A.V., Knapp S., Schiöth H.B. (2021). Trends in kinase drug discovery: Targets, indications and inhibitor design. Nat. Rev. Drug Discov..

[3] Amrhein J.A., Knapp S., Hanke T. (2021). Synthetic opportunities and challenges for macrocyclic kinase inhibitors. J. Med. Chem..

[4] Liang Y., Fang R., Rao Q. (2022). An Insight into the Medicinal Chemistry Perspective of Macrocyclic Derivatives with Antitumor Activity: A Systematic Review. Molecules.

[5] Giordanetto F., Kihlberg J. (2014). Macrocyclic drugs and clinical candidates: What can medicinal chemists learn from their properties?. J. Med. Chem..

[6] Sun D. (2022). Recent advances in macrocyclic drugs and microwave-assisted and/or solid-supported synthesis of macrocycles. Molecules.

[7] Yu X., Sun D. (2013). Macrocyclic drugs and synthetic methodologies toward macrocycles. Molecules.

[8] Marti-Centelles V., Pandey M.D., Burguete M.I., Luis S.V. (2015). Macrocyclization reactions: The importance of conformational, configurational, and template-induced preorganization. Chem. Rev..

[9] Itoh H., Inoue M. (2019). Comprehensive structure–activity relationship studies of macrocyclic natural products enabled by their total syntheses. Chem. Rev..

[10] Bhujbal S.P., Keretsu S., Balasubramanian P.K., Cho S.J. (2019). Macrocyclic effect on inhibitory activity: A modeling study on MerTK inhibitors. Med. Chem. Res..

[11] Marsault E., Peterson M.L. (2011). Macrocycles are great cycles: Applications, opportunities, and challenges of synthetic macrocycles in drug discovery. J. Med. Chem..

[12] Bridger G.J., Skerlj R.T., Thornton D., Padmanabhan S., Martellucci S.A., Henson G.W., Abrams M.J., Yamamoto N., Vreese K.D. (1995). Synthesis and structure-activity relationships of phenylenebis (methylene)-linked bis-tetraazamacrocycles that inhibit HIV replication. Effects of macrocyclic ring size and substituents on the aromatic linker. J. Med. Chem..

[13] Hawkins P.C. (2017). Conformation generation: The state of the art. J. Chem. Inf. Model..

[14] Mallinson J., Collins I. (2012). Macrocycles in new drug discovery. Futur. Med. Chem..

[15] DeLorbe J.E., Clements J.H., Whiddon B.B., Martin S.F. (2010). Thermodynamic and structural effects of macrocyclic constraints in protein-ligand interactions. ACS Med. Chem. Lett..

[16] Yu H.S., Deng Y., Wu Y., Sindhikara D., Rask A.R., Kimura T., Abel R., Wang L. (2017). Accurate and reliable prediction of the binding affinities of macrocycles to their protein targets. J. Chem. Theory Comput..

[17] Sánchez-Duffhues G., Williams E., Benderitter P., Orlova V., van Wijhe M., Garcia de Vinuesa A., Kerr G., Caradec J., Lodder K., de Boer H.C. (2019). Development of macrocycle kinase inhibitors for ALK2 using fibrodysplasia ossificans progressiva-derived endothelial cells. JBMR Plus.

[18] Reguera L., Rivera D.G. (2019). Multicomponent reaction toolbox for peptide macrocyclization and stapling. Chem. Rev..

[19] El Darsa H., Abdel-Rahman O., Sangha R. (2020). Pharmacological and clinical properties of lorlatinib in the treatment of ALK-rearranged advanced non-small cell lung cancer. Expert Opin. Pharmacother..

[20] Van de Weghe P., Eustache J. (2005). The application of olefin metathesis to the synthesis of biologically active macrocyclic agents. Curr. Top. Med. Chem..

[21] Ma J., Sanchez-Duffhues G., Caradec J., Benderitter P., Hoflack J., Dijke P.T. (2022). Development of small macrocyclic kinase inhibitors. Futur. Med. Chem..

[22] Farand J., Mai N., Chandrasekhar J., Newby Z.E., Van Veldhuizen J., Loyer-Drew J., Venkataramani C., Guerrero J., Kwok A., Li N. (2016). Selectivity switch between FAK and Pyk2: Macrocyclization of FAK inhibitors improves Pyk2 potency. Bioorganic Med. Chem. Lett..

[23] Cee V.J., Chavez F., Herberich B., Lanman B.A., Pettus L.H., Reed A.B., Wu B., Wurz R.P., Andrews K.L., Chen J. (2016). Discovery and optimization of macrocyclic quinoxaline-pyrrolo-dihydropiperidinones as potent pim-1/2 kinase inhibitors. ACS Med. Chem. Lett..

[24] Xu J., Shen C., Xie Y., Qiu B., Ren X., Zhou Y., Li G., Zheng G., Huang N. (2022). Design, synthesis, and bioactivity evaluation of macrocyclic benzo [b] pyrido [4, 3-e][1, 4] oxazine derivatives as novel Pim-1 kinase inhibitors. Bioorganic Med. Chem. Lett..

[25] Tao Z.-F., Wang L., Stewart K.D., Chen Z., Gu W., Bui M.-H., Merta P., Zhang H., Kovar P., Johnson E. (2007). Structure-based design, synthesis, and biological evaluation of potent and selective macrocyclic checkpoint kinase 1 inhibitors. J. Med. Chem..

[26] Lücking U., Siemeister G., Schäfer M., Briem H., Krüger M., Lienau P., Jautelat R. (2007). Macrocyclic aminopyrimidines as multitarget CDK and VEGF-R inhibitors with potent antiproliferative activities. ChemMedChem.

[27] Nie Z., Perretta C., Erickson P., Margosiak S., Lu J., Averill A., Almassy R., Chu S. (2008). Structure-based design and synthesis of novel macrocyclic pyrazolo [1,5-a][1,3,5] triazine compounds as potent inhibitors of protein kinase CK2 and their anticancer activities. Bioorganic Med. Chem. Lett..

[28] Johnson T.W., Richardson P.F., Bailey S., Brooun A., Burke B.J., Collins M.R., Cui J.J., Deal J.G., Deng Y.-L., Dinh D. (2014). Discovery of (10 R)-7-Amino-12-fluoro-2, 10, 16-trimethyl-15-oxo-10, 15, 16, 17-tetrahydro-2H-8, 4-(metheno) pyrazolo [4,3-h][2,5,11]-benzoxadiazacyclotetradecine-3-carbonitrile (PF-06463922), a macrocyclic inhibitor of anaplastic lymphoma kinase (ALK) and c-ros oncogene 1 (ROS1) with preclinical brain exposure and broad-spectrum potency against ALK-resistant mutations. J. Med. Chem..

[29] Engelhardt H., Böse D., Petronczki M., Scharn D., Bader G., Baum A., Bergner A., Chong E., Döbel S., Egger G. (2019). Start selective and rigidify: The discovery path toward a next generation of EGFR tyrosine kinase inhibitors. J. Med. Chem..

[30] Gajiwala K.S., Grodsky N., Bolaños B., Feng J., Ferre R., Timofeevski S., Xu M., Murray B.W., Johnson T.W., Stewart A. (2017). The Axl kinase domain in complex with a macrocyclic inhibitor offers first structural insights into an active TAM receptor kinase. J. Biol. Chem..

[31] Wang C., Li J., Qu L., Tang X., Song X., Yang F., Chen X., Lin Q., Lin W., Zhou Y. (2022). Discovery of D6808, a highly selective and potent macrocyclic c-met inhibitor for gastric cancer harboring MET gene alteration treatment. J. Med. Chem..

[32] Kurz C.G., Preuss F., Tjaden A., Cusack M., Amrhein J.A., Chatterjee D., Mathea S., Berger L.M., Berger B.-T., Kramer A. (2022). Illuminating the Dark: Highly Selective Inhibition of Serine/Threonine Kinase 17A with Pyrazolo [1,5-a] pyrimidine-Based Macrocycles. J. Med. Chem..

[33] Himmelbauer M.K., Xin Z., Jones J.H., Enyedy I., King K., Marcotte D.J., Murugan P., Santoro J.C., Hesson T., Spilker K. (2019). Rational design and optimization of a novel class of macrocyclic apoptosis signal-regulating kinase 1 inhibitors. J. Med. Chem..

[34] Driggers E.M., Hale S.P., Lee J., Terrett N.K. (2008). The exploration of macrocycles for drug discovery—An underexploited structural class. Nat. Rev. Drug Discov..

[35] Lee J.-S., Song I.-h., Shinde P.B., Nimse S.B. (2020). Macrocycles and supramolecules as antioxidants: Excellent scaffolds for development of potential therapeutic agents. Antioxidants.

[36] Roskoski R. (2021). Properties of FDA-approved small molecule protein kinase inhibitors: A 2021 update. Pharmacol. Res..

[37] Musi C.A., Agrò G., Santarella F., Iervasi E., Borsello T. (2020). JNK3 as therapeutic target and biomarker in neurodegenerative and neurodevelopmental brain diseases. Cells.

[38] Jun J., Yang S., Lee J., Moon H., Kim J., Jung H., Im D., Oh Y., Jang M., Cho H. (2023). Discovery of novel imidazole chemotypes as isoform-selective JNK3 inhibitors for the treatment of Alzheimer’s disease. Eur. J. Med. Chem..

[39] Jun J., Moon H., Yang S., Lee J., Baek J., Kim H., Cho H., Hwang K., Ahn S., Kim Y. (2023). Carbamate JNK3 Inhibitors Show Promise as Effective Treatments for Alzheimer’s Disease: In Vivo Studies on Mouse Models. J. Med. Chem..

[40] Choi K.-E., Balupuri A., Kang N.S. (2023). TWN-RENCOD: A novel method for protein binding site comparison. Comput. Struct. Biotechnol. J..

[41] Govindaraj R.G., Brylinski M. (2018). Comparative assessment of strategies to identify similar ligand-binding pockets in proteins. BMC Bioinform..

[42] Volkamer A., Rarey M. (2014). Exploiting structural information for drug-target assessment. Futur. Med. Chem..

[43] Ehrt C., Brinkjost T., Koch O. (2018). A benchmark driven guide to binding site comparison: An exhaustive evaluation using tailor-made data sets (ProSPECCTs). PLoS Comput. Biol..

[44] Halgren T.A. (2009). Identifying and characterizing binding sites and assessing druggability. J. Chem. Inf. Model..

[45] Halgren T. (2007). New method for fast and accurate binding-site identification and analysis. Chem. Biol. Drug Des..

